# A PCR-Based Method to Construct Lentiviral Vector Expressing Double Tough Decoy for miRNA Inhibition

**DOI:** 10.1371/journal.pone.0143864

**Published:** 2015-12-01

**Authors:** Huiling Qiu, Jiasheng Zhong, Lan Luo, Nian Liu, Kang Kang, Junle Qu, Wenda Peng, Deming Gou

**Affiliations:** 1 Shenzhen Key Laboratory of Microbial Genetic Engineering, College of Life Sciences, Shenzhen University, Shenzhen, Guangdong, 518060, China; 2 Key Laboratory of Optoelectronic Devices and Systems of Ministry of Education and Guangdong Province, College of Optoelectronic Engineering, Shenzhen University, Shenzhen, Guangdong, 518060, China; 3 Department of Physiology, Shenzhen University Health Science Center, Shenzhen, Guangdong, 518000, China; French National Center for Scientific Research - Institut de biologie moléculaire et cellulaire, FRANCE

## Abstract

DNA vector-encoded Tough Decoy (TuD) miRNA inhibitor is attracting increased attention due to its high efficiency in miRNA suppression. The current methods used to construct TuD vectors are based on synthesizing long oligonucleotides (~90 mer), which have been costly and problematic because of mutations during synthesis. In this study, we report a PCR-based method for the generation of double Tough Decoy (dTuD) vector in which only two sets of shorter oligonucleotides (< 60 mer) were used. Different approaches were employed to test the inhibitory potency of dTuDs. We demonstrated that dTuD is the most efficient method in miRNA inhibition *in vitro* and *in vivo*. Using this method, a mini dTuD library against 88 human miRNAs was constructed and used for a high-throughput screening (HTS) of AP-1 pathway-related miRNAs. Seven miRNAs (miR-18b-5p, -101-3p, -148b-3p, -130b-3p, -186-3p, -187-3p and -1324) were identified as candidates involved in AP-1 pathway regulation. This novel method allows for an accurate and cost-effective generation of dTuD miRNA inhibitor, providing a powerful tool for efficient miRNA suppression *in vitro* and *in vivo*.

## Introduction

MicroRNAs (miRNAs) are small non-coding RNAs that post-transcriptionally control gene expression. It is thought that miRNAs bind to target mRNA through specific base pairing, causing a translational repression and/or degradation of targeting mRNA [1]. miRNAs modulate almost all biological processes, including cell proliferation, differentiation, apoptosis, signaling, stress response and organ development [2], but only a small number of miRNAs have been experimentally validated. Growing evidence demonstrates that many diseases in humans could be caused by aberrant expression of miRNAs [3–5]. Therefore, methods in manipulating miRNA expression levels are necessary to dissect the function of miRNAs and to explore their therapeutic applications.

Currently, there are two major types of miRNA inhibitors: chemically synthesized and DNA vector-expressed. Initially, inhibition of miRNA activity was accomplished by delivering the synthetized antisense oligonucleotides, such as 2′-*O*-methyl RNA [6,7], locked nucleic acid and antagomirs [8,9]. However, this type of inhibition is transient and often toxic due to off-target effects. Long-term stable inhibition of miRNA is achieved by vector-encoded miRNA inhibitors, such as antagomirs [10], sponge [11], miRzip and Tough Decoy (TuD) [12]. In particular, the TuD approach has attracted more attention due to its specific and long-term inhibitory effect on miRNAs [12].

The TuD molecule has a complex structure consisting of a stem-loop harboring a loop composed of two miRNA binding sites (MBS) with four nucleotides mismatches insert, a flanked stem of 18 bp on MBS and four linkers with three nucleotides combining flanked stem [12]. So far, the TuD inserts are generated using two long synthesized oligonucleotides (~90 mer), which is costly and often problematic because of mutations during synthesis.

In this study, we developed a PCR-based method to construct lentiviral vector expressing double molecules TuD (dTuD). We demonstrated that dTuD suppressed target miRNAs specifically and efficiently *in vitro* and *in vivo*. Based on two-step PCR, we constructed a dTuD library and used to identify AP-1 pathway-related miRNAs by high-throughput screening (HTS). Our data demonstrated that the PCR-based method is a simple and cost-effective approach to generate dTuD lentiviral vector.

## Materials and Methods

### Cell culture and transfection

293A, 293T and C2C12 cells were purchased from American Type Culture Collection and maintained in Dulbecco’s modified Eagle’s medium supplemented with 10% fetal bovine serum in a humidified incubator with 5% CO_2_. The linear polyethylenimine was used as transfection reagent.

### PCR primer and conditions in generating dTuD vector

The primers used in the present study are shown in S1 Table and synthesized by Life Invitrogene. The PCR conditions were as followed: initial denature at 95°C for 2 min, 3 cycles of 95°C for 30 s, 52°C for 30 s and 72°C for 1 min, 25 cycles of 95°C for 30 s, 68°C for 1 min.

### Luciferase reporter assay

The 3′UTR of human RhoB containing miR-223 binding sites were amplified by PCR using primers of RhoB-F (5′- GTCGAATTCAGCAAGCCACTACTGTTGCTCCATG -3′) and RhoB-R (5′- AGCTCTCGAGTCTTCTGACACTATTAAGCCACAGG -3′) and subcloned into the pmirGLO vector through *Eco*RI-*Xho*I sites (Promega, Madison, WI), resulting in the pmirGLO-RhoB 3′UTR vector. For reporter assays, 1×10^4^ 293A cells overnight cultured on 48-well plates were transfected with a mixture of pmirGLO-RhoB 3′UTR reporter (50 ng), miR-223 mimics (100 μM) and dTuD-miR-223 (300 ng), and harvested 48h after transfection. The luciferase activities were measured on Lumat3 (Berthold technologies, LB9508) with the Dual-luciferase Reporter Assay System (Promega, Madison, WI) according to manufacturer’s instructions. For Activator Protein 1 (AP-1) pathway reporter assays, 293A cells cultured on 96-well plates were transfected with a mixture of AP-1 reporter (50 ng, Clontech), phRL-SV40 (1 ng, Promega) and dTuD (150 ng), and further divided into two groups with or without the treatment of phorbol 12-myristate 13-acetate (PMA, Sigma, St Louis, MO). For the PMA stimulation group, 24 h after transfection, 293A cells were treated with PMA (10 ng/mL) for 18h. 293A cells with or without PMA stimulation were harvested for luciferase activity measurement.

### Western blot analysis

The total proteins were extracted with mRIPA mammalian protein extraction lysis buffer (50 mM Tris-HCl, 150 mM NaCl, 1% NP-40, 0.25% sodium deoxycholate, and 1 mM EDTA) supplemented with protease inhibitor cocktail (Roche, Mannheim, Germany). Primary antibodies used are as follows: anti-CDC25A (1:1000 dilution, Santa Cruz), anti-CCNE1 (1:1000 dilution, Santa Cruz), anti-myogenin (1:2000 dilution, Proteintech) and anti-β-actin (1:10000 dilution, Proteintech).

### Lentivirus packaging, titration and transduction

High titer dTuD lentivirus particles were packaged in 293T cells by transfection of three individual plasmids at a 2:1:5 ratios: (1) psPAX2 plasmid encoding HIV Gag-Pol (Addgene, 12260); (2) pVSVG (Addgene, 8454), encoding the VSV-G glycoprotein; and (3) the dTuD lentiviral vector. Briefly, 293T cells were seeded on 10 cm culture plates at a density of 4×10^6^ cells per plate. After 24h incubation, cells were transfected with lentiviral vector (12.5 μg) and packing plasmids (7.5 μg) using polyethylenimine reagent. Cell-free supernatants were harvested 48 and 72h after transfection and used for subsequent cell transductions in the presence of 6 ng/ml polybrene. Successfully transduced cells were selected by supplementing the culture medium with 1.0–2.0 μg/ml puromycin after 48h transduction.

### RNA extraction and quantitative RT-PCR analysis

Total RNA was extracted with RNAiso Reagent (TaKaRa, Dalian, China) according to manufacturer’s instructions. The expression of mature miRNA was measured by our previously developed S-poly (T) miRNA method [13], using snoRNA 44 and snoRNA 234 as human and mouse miRNA endogenous reference, respectively. PCR was performed using Step One Plus system (Applied Biosystem, Foster City, CA). The data was normalized to that of endogenous reference and calculated using the 2^-ΔΔ^CT method.

### Ethics statements

The use of animals was in accordance with the recommendations in the Guide for the Care and Use of Laboratory Animals of China; it was approved by the Animal Ethical and Welfare Committee of Shenzhen University (Approval No. AEWC-2014-001004). All treatments were performed under nembutal anesthesia to minimize suffering.

### Mice and *in vivo* transfection

BALB/c mice were purchased from Guangdong Medical laboratory Animal Center. Two-week-old male mice were used for the experiment. Mice were anesthetized by intraperitoneal injection of 1% nembutal at 50 mg/g. Mice were injected intraperitoneally 2.5 μg of dTuDs with *In vivo* jetPEI DNA Transfection Reagent (Polyplus Transfection, New York, NY.) every two weeks, three injections in total. Mice were sacrificed two weeks after the last injection and gastrocnemius muscles were collected for RNA and protein analysis.

## Results

### Construct dTuD lentiviral vector based on two-step PCR

**Fig 1:**
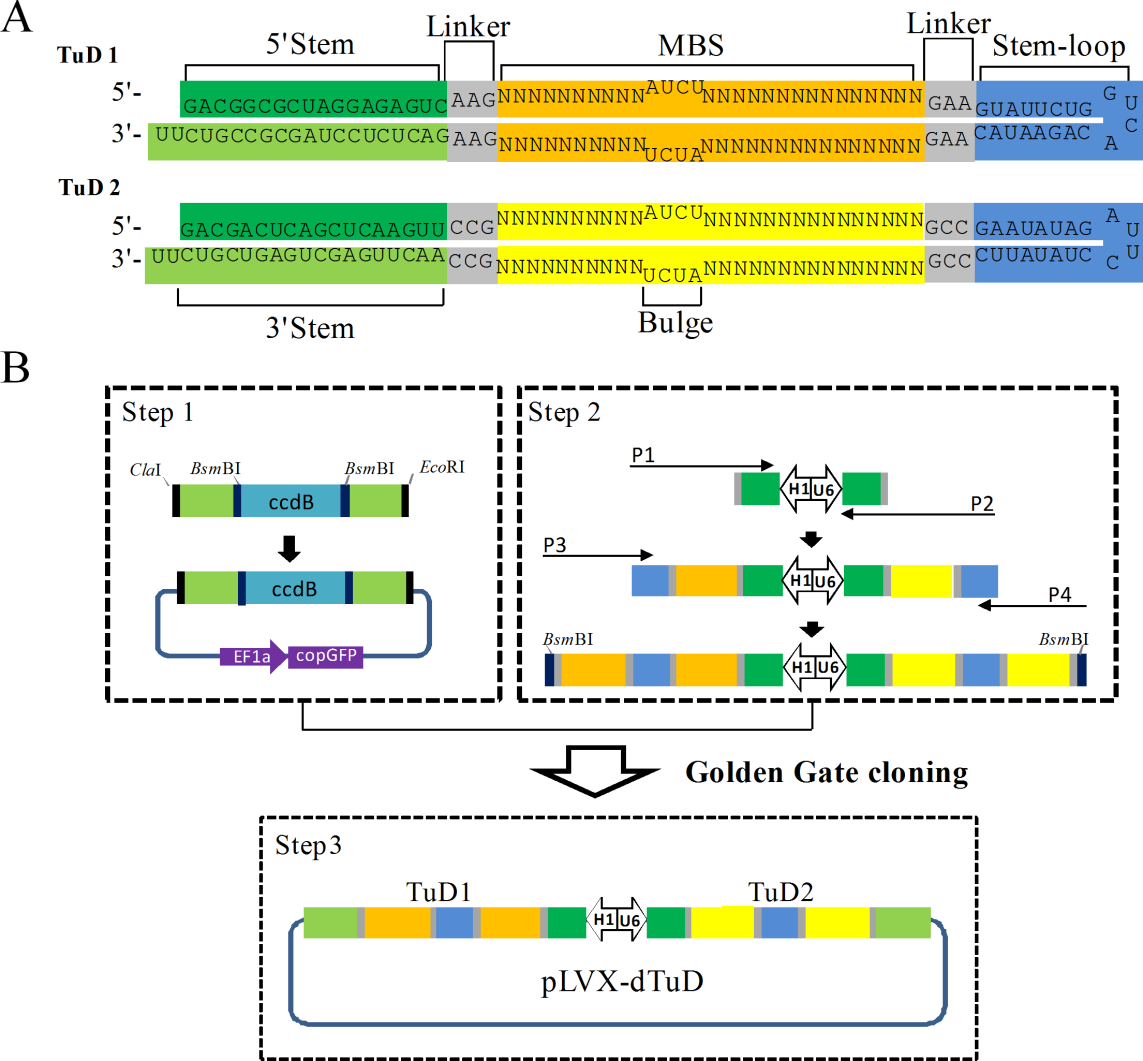
Schematic outline for the construction of pLVX-dTuD vector. **(A)** Overview of a dTuD lentivirus-based vector (pLVX-dTuD) encoding two TuD RNA molecules. Each TuD RNA includes one stem (18 nt), two MBS with bulge (4 nt), one stem-loop (10 nt) and four linkers (3 nt) between stem and MBS. **(B)** Strategy to construct pLVX-dTuD vector. Step 1: preparation of the MBS-recipient vector. Step 2: preparation of MBS-donor by two-step PCR. Step 3: MBS-donor fragment was inserted into MBS-recipient vector by utilizing the Golden Gate cloning.

It has been demonstrated that lentiviral vector expressing TuD can achieve long-term suppression of miRNA [12]. A single vector expressing multiple decoy RNAs can improve inhibitory efficiency [14,15]. In order to increase the TuD expression level for better miRNA suppression, we designed a lentiviral vector harboring two TuD expression cassettes driven by our previously reported H1-U6 bidirectional promoter [16], and designated it as dTuD. Fig 1A shows each TuD sequences encoded by dTuD lentiviral vector, which is constructed by a PCR-based method. The detail procedure is described by the following three steps (Fig 1B).

#### Step 1: preparation of MBS-recipient vector

We used the pLVX-Puro vector (Clontech) as a backbone vector. There are two *Bsm*BI sites within PGK promoter and puromycin resistant selection gene. We first performed the mutagenesis to remove both *Bsm*BI sites using overlap PCR strategy. The *Bsm*BI site in PGK promoter was changed to CGCCTC but did not affect promoter activity (data not shown), while the *Bsm*BI site in puromycin resistant selection gene was changed to CGTGTC without affecting the encoded amino acid. Then, the PCR product of EF1α promoter-driven copGFP was cloned into the modified pLVX-Puro vector, named pLVX-copGFP. Finally, pLVX-copGFP was digested with *Cla*I-*Eco*RI and inserted a PCR fragment harboring *Cla*I-3′stem-*Bsm*BI-ccdB-*Bsm*BI-3′stem-*Eco*RI (S2 Table), resulting in MBS-recipient vector. Since this vector has a negative selection marker of ccdB, the positive clones with MBS-donor inserts will survive in E.Coli STBL3.

#### Step 2: preparation of MBS-donor fragments

To prepare the MBS-donor fragments, we first generated a universal PCR template that included a bidirectional H1-U6 promoter flanked by 5′stem and linker sequences. Then, two sets of primers designed by our Excel-based program dTuD_Primer_Design (S1 File) were used for the two-step PCR amplification. In the dTuD_Primer_Design program, (1) the mature miRNA sequences is inputted, ‘U’ is automatically replaced by ‘T’, (2) four nucleotides of ‘ATCT’ are added between nucleotides 10 and 11 from the 5′end, named sense MBS, (3) the modified sense MBS is converted to anti-sense MBS, and (4) four primers are generated as followed: P1 (5′-GTATTCTGTGACCAGAATACTTC-sense MBS-CTTGACTCTC-3′), P2 (5′-GAATATAGGAATCTATATTCGGC-anti-sense MBS-CGGAACTTGA-3′), P3 (5′-ACCCGTCTCTCTTC-sense MBS-CTTGTATTCTGTGACCAGA-3′) and P4 (5′-GTGCGTCTCATGGC-anti-sense MBS-CGGGAATATAGGAATCTAT-3′). Using the pre-prepared bidirectional H1-U6 promoter flanked by 5′stem and linker sequences as template, the first step in PCR is P1 and P2 primers amplification followed by P3 and P4 primers amplification.

#### Step 3: assembly of MBS-donor and MBS-recipient vector by Golden Gate cloning

We assembled the MBS-donor fragment and MBS-recipient vector together by Golden Gate cloning. The negative selection marker of ccdB allowed a zero background in cloning and a copGFP reporter help to track the transduction efficiency.

### Dose-dependent increase of miRNA suppression by dTuD

**Fig 2:**
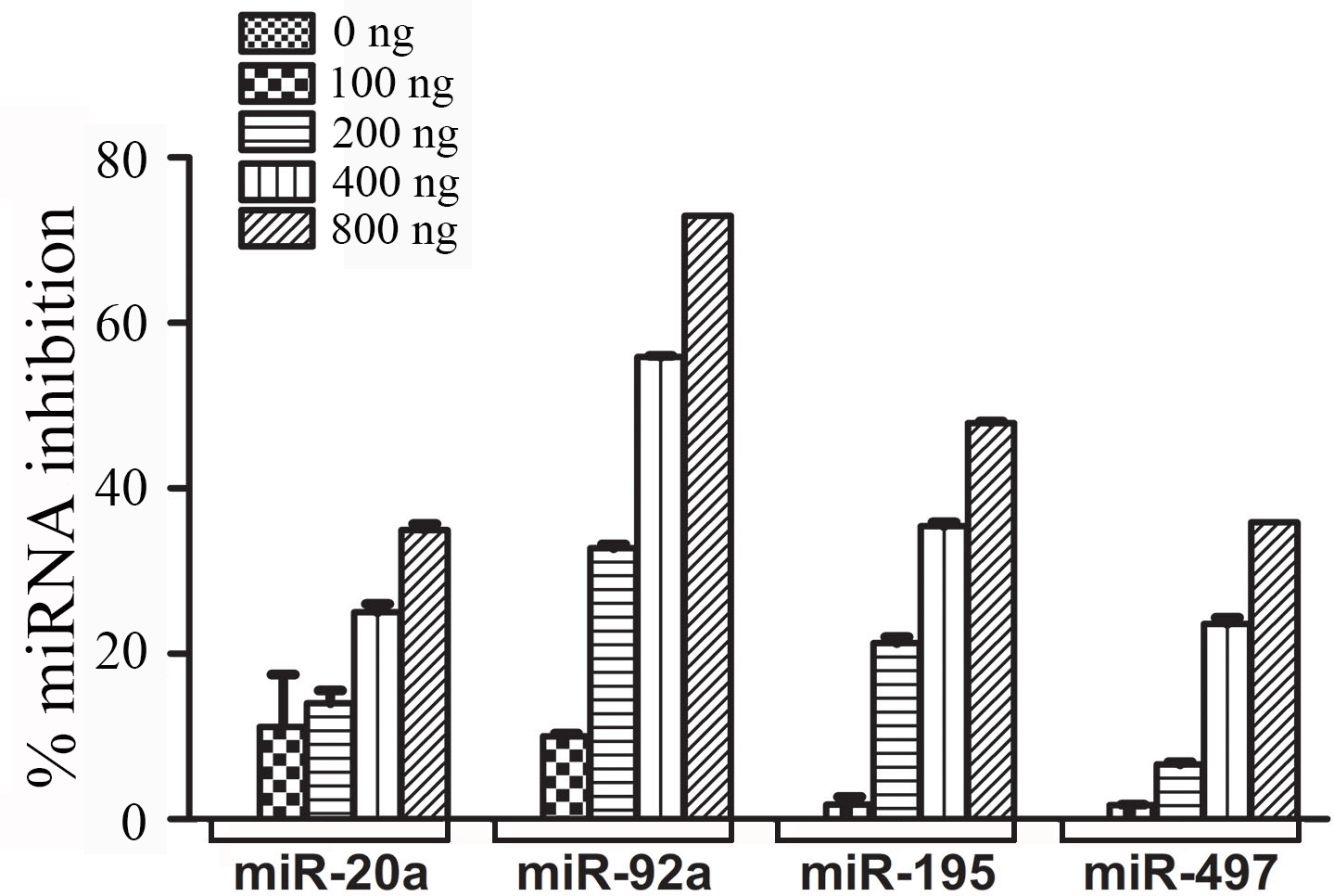
Dose-dependency of dTuD on the expression of related miRNAs. 293A cells were transfected with dTuD targeting miR-20a, -92a, -195 or -497 at the concentration of 0, 100, 200, 400 and 800 ng/mL. qRT-PCR was performed to evaluate the expression of miRNA. Data was shown as the percentage of miRNA in the cells transfected with dTuD via that in the cells transfected with dTuD-Ctrl. Data were represented as the mean ± SD (n = 3).

Previous studies have demonstrated that qRT-PCR can be used to evaluate the inhibitory efficiency of miRNA inhibitor [10,12,17,18]. Here, we selected four highly expressed miRNAs in 293A cells (miR-20a, -92a, -195 and -497) (S1 Fig) and determined the miRNA inhibition efficiency by transfecting various amount of each corresponding dTuD vector. As shown in Fig 2, the proportion of miRNAs inhibition was coupled with increased amount of dTuDs in a dose-dependent manner. 35%, 73%, 53% and 35% of miR-20a, -92a, -195 and -497 were efficiently inhibited by each corresponding dTuD at the concentration of 800 ng/mL.

### High specificity of dTuD in suppressing miRNA

**Fig 3:**
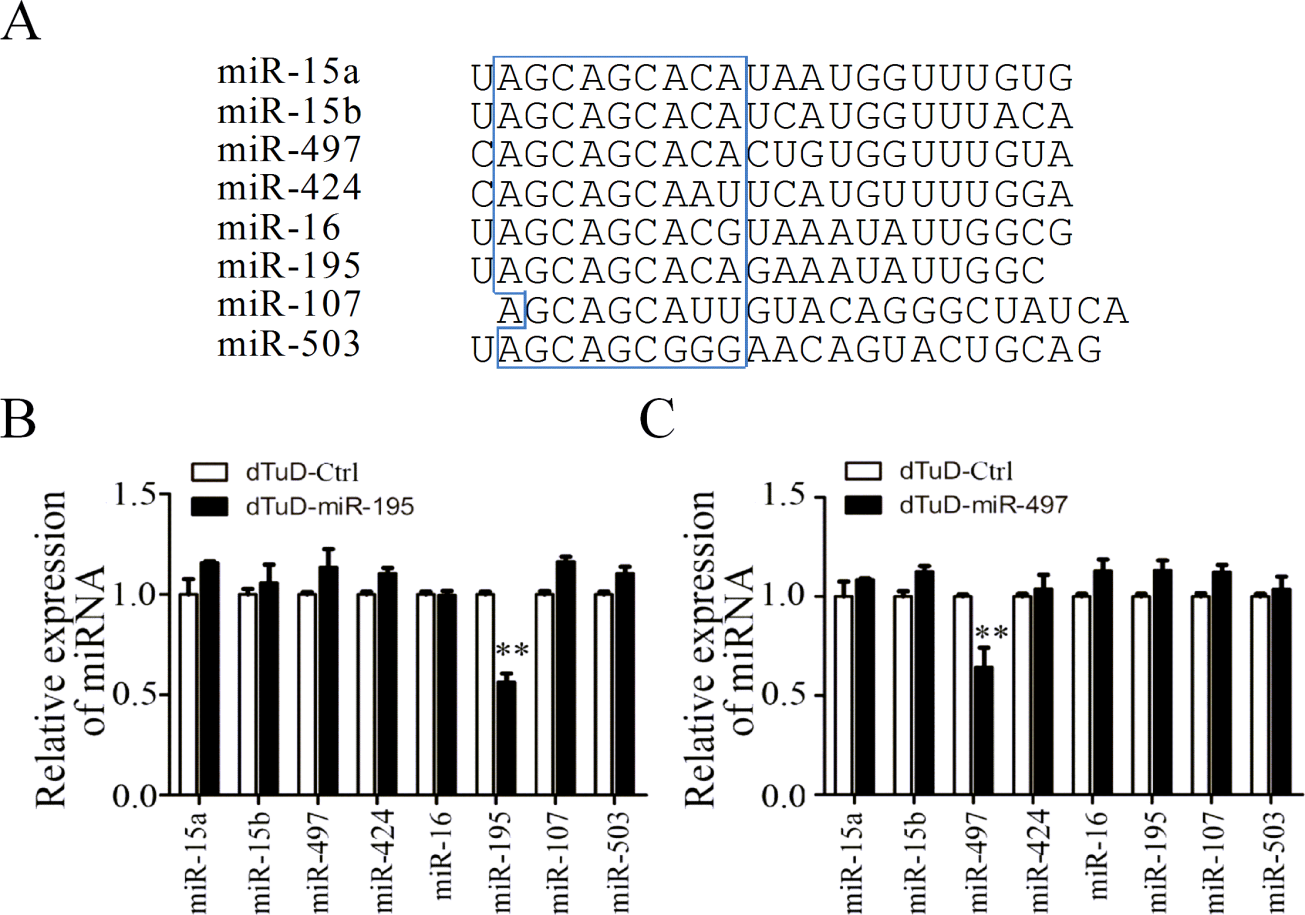
Specificity of dTuD on related miRNAs. (A) miR-15a, -15b, -16, -107, -195, -424, -497 and -503 sequences. Seed sequences are shown in box. (B-C) The expression levels of indicated miRNAs in the 293A cells transfected with dTuD-miR-195 (B) or dTuD-miR-497 (C) were evaluated 48h after the transfection by qRT-PCR. The expression of miRNA was normalized to that of dTuD-Ctrl transfected cells. Data were represented as the mean ± SD (n = 3). ** *p* < 0.01.

We next investigated the specificity of dTuD by examining the impact of dTuD suppressing miRNAs with conserved sequences. We chose one well-studied miRNA cluster, miR-16 cluster, which is comprised of seven members of miR-15a, -15b, -16, -107, -195, -424, -497 and -503 with similar seed sequences and highly expressed in 293A cells (Fig 3A and S2 Fig). We transfected dTuD-miR-195 or dTuD-miR-497 into 293A cells respectively and then measured the expression level of each miRNA in this cluster. We observed that only miR-195 or miR-497 was significantly decreased in cells transduced with corresponding dTuD, whereas no significant effects were found on other family members (Fig 3B and 3C). These results demonstrated that dTuD specifically reduced the target miRNA level without suppressing other miRNAs even when they share the same seed sequence.

### dTuD vector is more effective than TuD vector in miRNA inhibition

**Fig 4:**
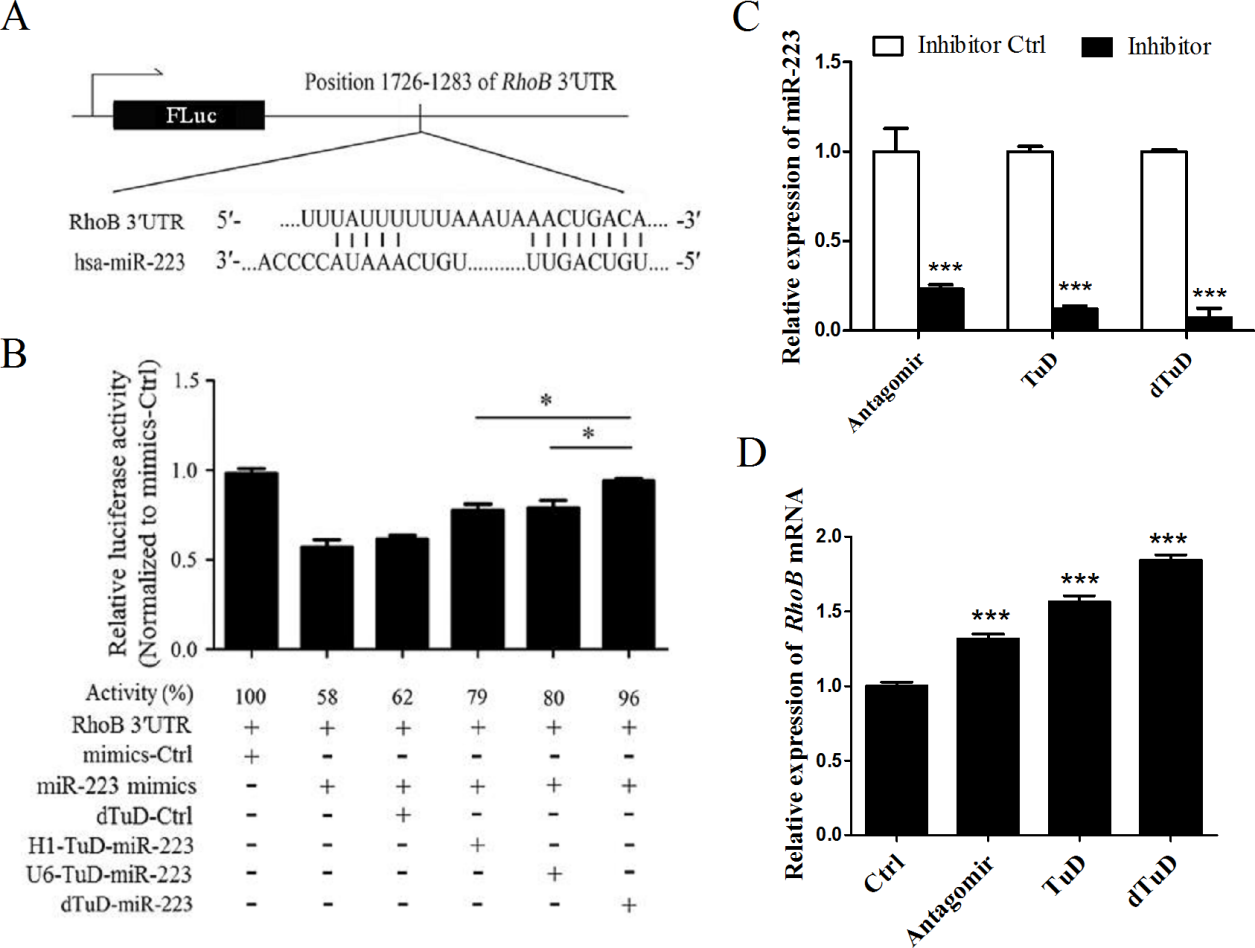
The inhibitory effect on miR-223 activity by dTuD-miR-223. (A) Schematic representation of 3′UTR luciferase reporter plasmid in which the 3′UTR of RhoB contained the binding sites of miR-233 (1726–1283 bp from 5′end) was fused to the 3′end of *Firefly Luciferase* (FLuc) after stop codon. (B) Dual-luciferase assay in 293A cells co-transfected with multiple plasmids as indicated. H1-TuD-miR-223 and U6-TuD-miR-223 represent the modified dTuD vector harboring a single TuD-miR-223 expression cassette driven by H1 and U6 promoter, respectively. After performing dual luciferase assay, the ratio FLuc/*Renilla* Luc (RLuc) was normalized to that of dTuD-Ctrl. (C) Expression level of miR-223 or (D) *RhoB* mRNA in 293A cells transfected with antagomir, TuD or dTuD against miR-233. Data were represented as the mean ± SD (n = 3). * p < 0.05, *** *p* < 0.001.

It has been reported that *RhoB* is the direct target of miR-223[19], therefore we generated a RhoB 3′UTR dual-luciferase reporter which contained a validated miR-223 binding sites (Fig 4A). We expected that the activity of luciferase could reflect the expression level of miR-223 in cells. To address whether dTuD vector is more effective in miRNA inhibition than the vector expressing a single TuD, we constructed dTuD-miR-223 against miR-223 and dTuD-Ctrl against scramble sequences. Two additional vectors, H1-TuD-miR-223 and U6-TuD-miR-223 expressing single TuD under control of H1 or U6 promoter were also constructed. As expected, the co-transfection of miR-223 mimics in 293A cells along with RhoB 3′UTR reporter plasmid suppressed luciferase activity by ~40% compared to mimics-Ctrl. The co-transfection of H1-TuD-miR-223 or U6-TuD-miR-223 suppressed luciferase activity by ~20%. The luciferase activities were almost attenuated by the co-transfected dTuD-miR-223, but not dTuD-Ctrl (Fig 4B). These data suggested that dTuD vector is better than TuD vector in miRNA inhibition. We next tested whether dTuD is still better than TuD in suppressing the activity of endogenous miRNA *in vitro*. We transfected the 293A cells with three types of anti-miR-223 inhibitors. Transfection of antagomir, TuD and dTuD reduced 77%, 88% and 93% (*p* < 0.001) of miR-223, respectively (Fig 4C) and depressed 32%, 57% and 84% (*p* < 0.001) of *RohB* mRNA (Fig 4D), showing that dTuD-miR-223 has great inhibitory effect on miR-223.

**Fig 5:**
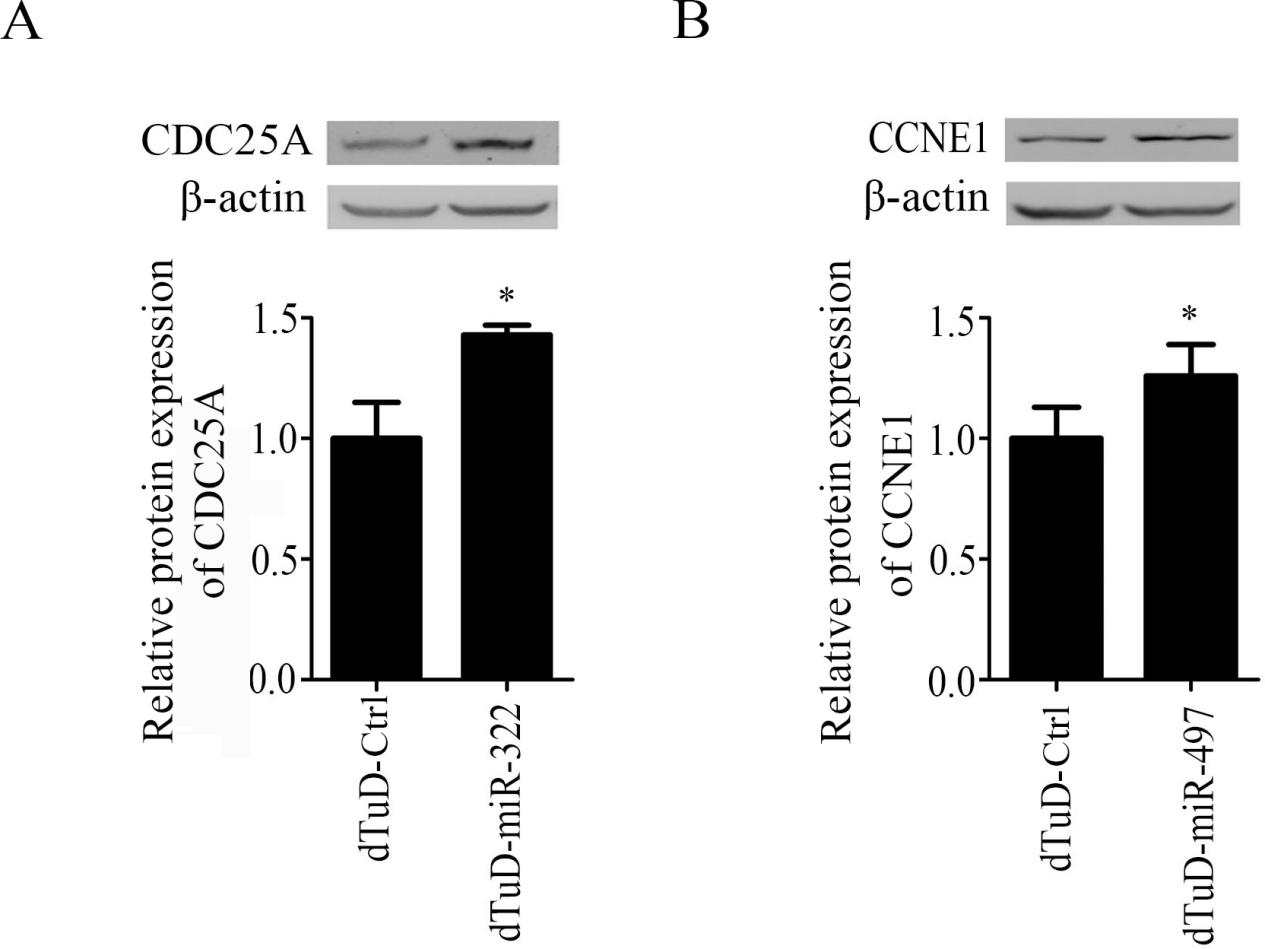
Inhibitory effects of dTuDs on protein expression of miRNA target. The protein expression level of CDC25A (A) or CCNE1 (B) in C2C12 cells transduced with lentivirus expressing dTuD-miR-322 or dTuD-miR-497, respectively. *CDC25A* and *CCNE1* are validated target genes of miR-322 and -497, respectively. The protein expression level was normalized to that of dTuD-Ctrl. Data were represented as the mean ± SD (n = 3).* p < 0.05.

Previous studies have confirmed that the miR-424 and miR-497 restrain cell proliferation by targeting human cell division cycle 25 homolog A (CDC25A) and G1/S-specific cycllin-E1 (CCNE1), respectively [20,21]. The rodent homologue of human miR-424 is mmu-miR-322. We transduced the C2C12 cells with dTuD lentivirus targeting miR-322, miR-497 or no-specific control sequences (dTuD-Ctrl). The transduction of dTuD-miR-322 and dTuD-miR-497 significantly increased the mRNA levels of target *CDC25A* (40%, *p* < 0.001) and *CCNE1* (57%, *p* < 0.001) (S3 Fig). Western blot showed that CDC25A and CCNE1 increased by 43% and 37% in dTuD-miR-322 and dTuD-miR-497 transduced cells, respectively, as compared to dTuD-Ctrl (Fig 5A and 5B). From these results, we can conclude that our dTuDs can lentivirally function *in vitro* and effectively suppress the endogenous target miRNAs, which subsequently attenuated the repressive effect of miRNA on target gene expression.

### dTuD functions efficiently *in vivo*

**Fig 6:**
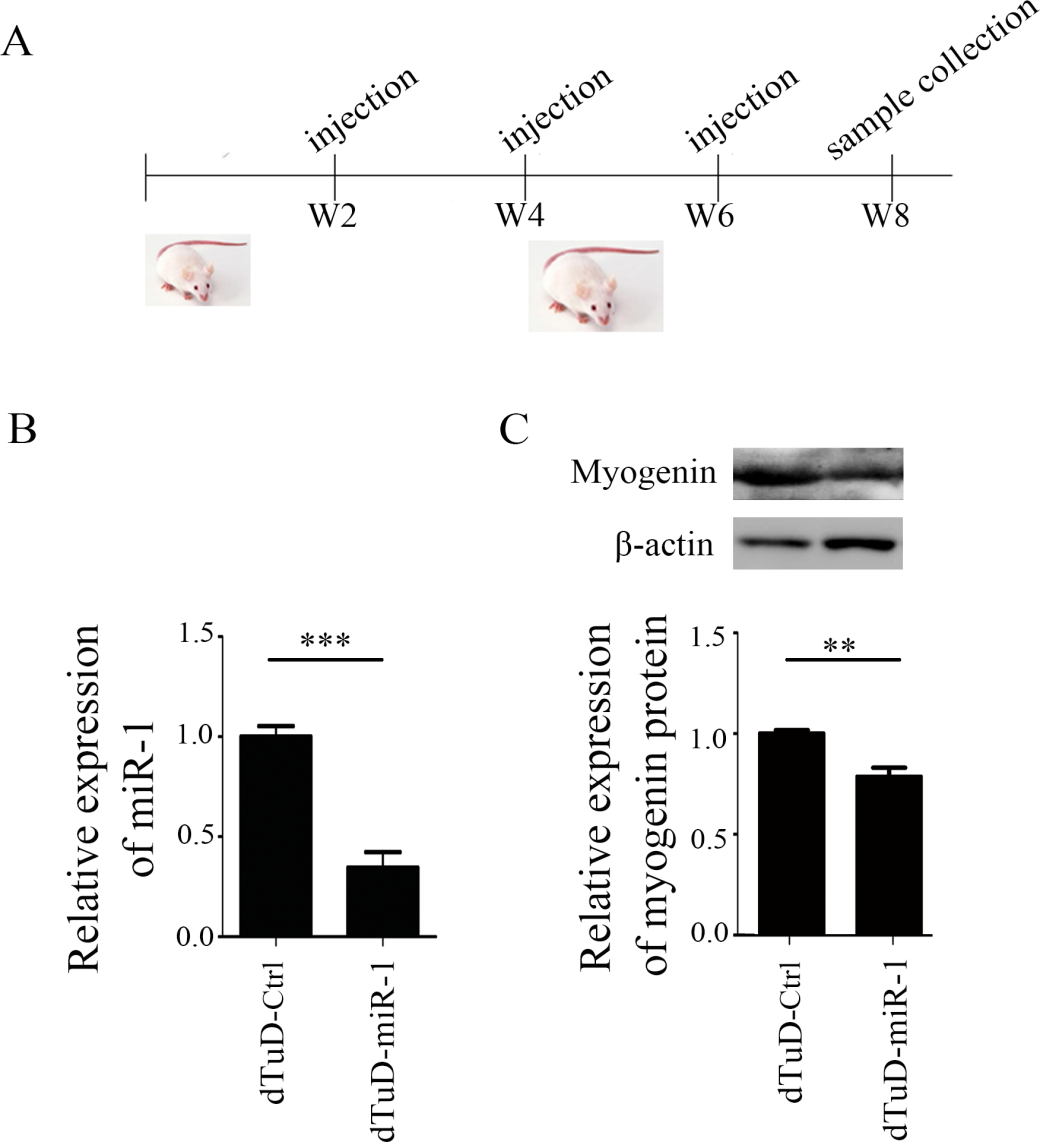
Inhibitory effect of dTuD-miR-1 in mice. (A) Schematic representation of dTuD-miR-1 intraperitoneal administration into mice. Two-week-old BALB/c mice were used, and received an intraperitoneal injection of dTuD every two weeks. Gastrocnemius muscles were collected two weeks after the third injection. (B) The expression of miR-1 in gastrocnemius muscle was measured by qRT-PCR. (C) The protein of myogenin, one of the miR-1 down-stream targets, was evaluated by Western blot. The expression level of miR-1 or myogenin was normalized to that of dTuD-Ctrl. Data were represented as the mean ± SD (n = 4) and compared between groups using the two-tailed Student’s *t*-test. ** *p <* 0.01, *** *p <* 0.001.

miR-1, a master myogenic miRNA, is required for myogenesis during skeletal muscle development by inducing the expression of myogenic regulatory factors, such as myoD and myogenin [22–24]. To test the effect of dTuD *in vivo*, dTuD against miR-1 (dTuD-miR-1) and scramble control (dTuD-Ctrl) were administrated to mice by intraperitoneal injection every two weeks, in total three injections (Fig 6A). We observed that both miR-1 (63%, *p* < 0.001) and myogenin protein (34%, *p* < 0.01) were significantly reduced in gastrocnemius muscles two weeks after the last injection (Fig 6B and 6C). There were no significant (*p* > 0.05) changes in body weight that received the dTuD-miR-1 (S4 Fig).

### High-throughput screening of AP-1 pathway-related miRNAs via dTuD library

**Fig 7:**
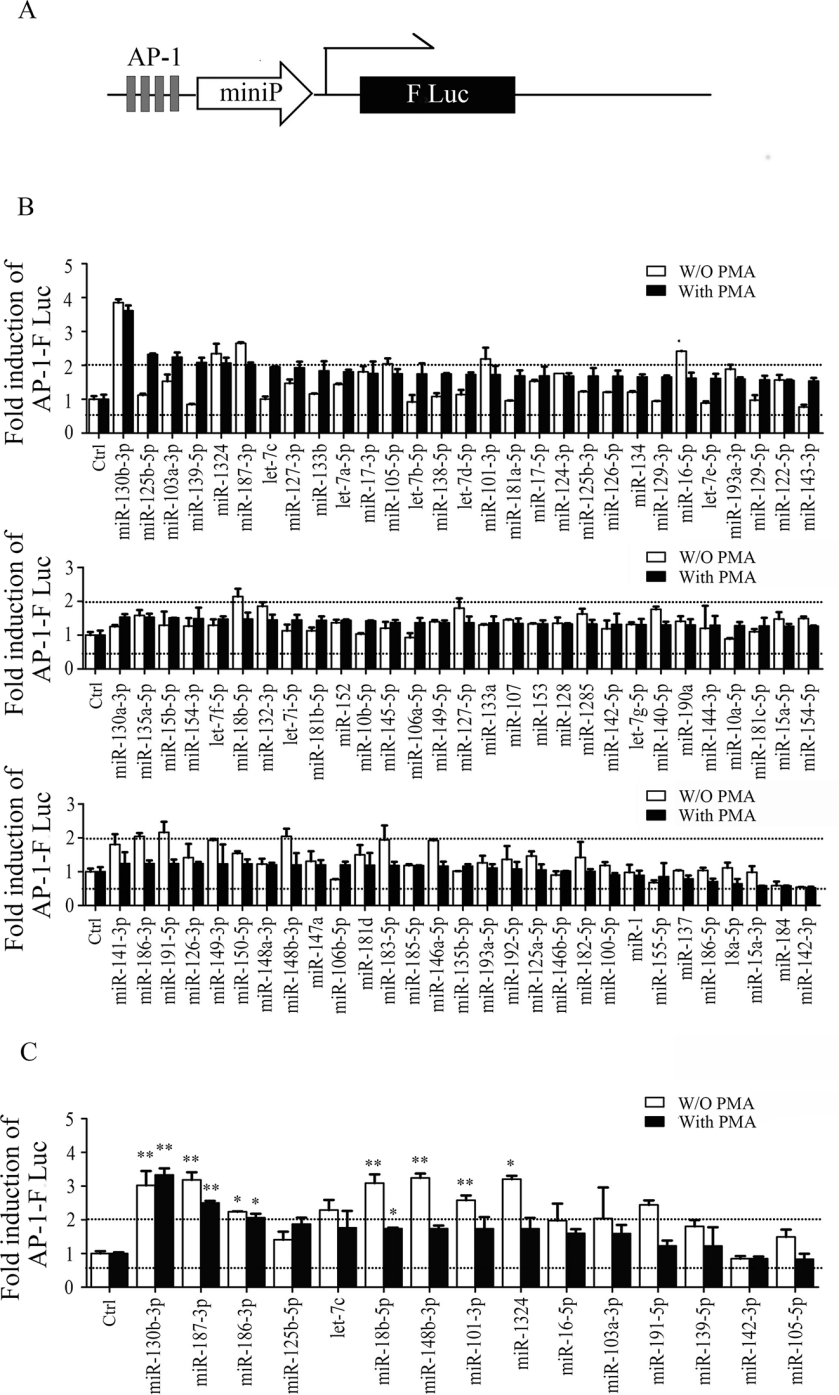
High-throughput screening for miRNAs involved in AP-1 pathway by dTuDs library and AP-1 luciferase reporter system. (A) Schematic representation of AP-1 indicator-sensor plasmid. (B-C) 293A cells were transfected with a mixture of three vectors: AP-1 luciferase reporter plasmid, RLuc plasmid and each dTuD vector. 24h after transfection, cells were treated with (black bars) or without (open bars) PMA for 18h and then harvested for luciferase activity assays. Relative activity of AP-1 were represented as the ratio of FLuc/RLuc normalized to that in cells transfected with dTuD-Ctrl. dTuDs targeting 88 miRNAs were tested for the first screen (B), and those miRNAs with a fold change > 2-fold were further verified following the same procedure as the first screen (C). The dashed lines indicate 2.0 fold above and 50% below the dTuD-Ctrl. Data were represented as the mean ± SD (n = 3). * *p* < 0.05, ** *p* < 0.01.

Based on this method, we generated a mini dTuDs library against 88 human primary miRNAs and applied to identify potential miRNAs involved in AP-1 signaling pathway, which can regulate cell proliferation, neoplastic transformation and apoptosis [25]. 293A cells cultured on 96-well plates were co-transfected with each dTuD vector targeting different miRNA along with AP-1 reporter and normalization control plasmid expressing *Renilla* luciferase (RLuc). Each transfection was executed in triplicate with or without the treatment of PMA, an AP-1 pathway activator. We identified a hit as the dTuD with a luciferase activity altered by more than 2-fold (2.0 fold above or 50% blow than dTuD-Ctrl). As shown in Fig 7, 15 dTuDs altered the luciferase activity under stimulation and/or un-stimulation of PMA (dTuD-miR-130b-3p, -125b-5p, -103a-3p, -139-5p, -1324, -187-3p, -101-3p, -16-5p, -18b-5p, -186-3p, -191-5p, -148b-3p and -142-3p). A second screen has been performed with these 15 dTuDs to confirm the results. Using the same criteria, we found that luciferase activity was up-regulated by four dTuDs (dTuD-miR-18b-5p, -148b-3p, -101-3p and -1324) under PMA stimulated but not un-stimulated condition, and three dTuDs (dTuD-miR-130b-3p, -186-3p and -187-3p) under both conditions. Therefore, these seven miRNAs can be identified as potential miRNAs involved in AP-1 pathway regulation.

## Discussion

In the present study, we describe a PCR-based method to construct lentiviral vector expressing double TuD at a greatly reduced cost with high efficacy. We estimate that this method could save over 50% of money from the synthesis of primers and DNA sequencing. It is extremely useful in generating dTuD library at a genome scale. Several groups have demonstrated that TuD is the most effective miRNA inhibitors *in* vitro and in vivo [17,26,27]. Due to the increase of miRNA binding sites, our data supported that dTuDs show stronger inhibitory activity than single TuD in miRNA inhibition (Fig 4), proving evidence that bidirectional promoter guarantees high expression of dTuD under the control of H1 and U6 promoters. Hollensen and colleagues have developed clustered TuD inhibitors [15]. However, the clustered TuDs are driven by a single PGK promoter, which cannot guarantee the exact transcriptional initiation and termination with limited transcriptional levels.

To reduce the size of oligonucleotides for PCR amplification, the conserved 5′stem and linker sequences were fused to the end of H1-U6 fragments by PCR primer extension. Similarly, the conserved 3′stem and linker sequences were pre-cloned into MBS-recipient vector. Two *Bsm*BI restriction sites between the linker sequences were created for the ligation of MBS-donor inserts. In order to improve cloning efficiency, a negative selection marker of ccdB was inserted between two *Bsm*BI restriction sites. In this case, only the positive clones with MBS-donor inserts survived in E.Coli STBL3, allowing zero background during cloning. With two sets of shorter primers, dTuD lentiviral vector can be constructed by two-step PCR and Golden Gate cloning strategy at a greatly reduced cost with high efficacy. We estimate that this method could save over 50% of money but double the inhibition efficacy.

Our results demonstrate that transfection or transduction of dTuDs can efficiently attenuated the activities of target miRNAs (Figs 4 and 5), which indicates that the vector structure can produce functional TuD molecules accurately. In our study, dTuD-miR-195 and dTuD-miR-497 repressed specifically target miRNAs but not the other miRNA family members (Fig 3), indicating that cross-reactivity among different miRNA family members was not detectable. In addition, the expression levels of the other family members were slightly increased (Fig 3), possibly reflecting feed-back regulation and functional overlap or compensation mechanisms among miRNAs with similar seed sequences. One such example is well represented by polycitronic miR-17-92 miRNA cluster [28].

The expression of miR-20a, 92a and 195 were at nearly the same level in 293A cells (S1 Fig). However, the levels of suppression were quite different among these miRNAs in the cells transfected with the same amount of corresponding dTuD (Fig 2), suggesting that the inhibitory effect of dTuD could vary from miRNA to miRNA. This could be ascribed to differences in the accessibility of TuDs to target miRNAs. It has been discussed that the inhibitory potency of TuD is probably the combined result of specific interaction between MBS and miRNA and base pairing between MBS in TuD hairpin [15].

miR-1 and -206 are well-established myogenesis and play critical roles in muscle differentiation [24,29]. In our study, knockdown of miR-1 in mice did not lead to any defect. It has been reported that even knockout of miR-1 in mice shows no apparent phenotypic consequences in skeletal muscle [30]. Similar to miR-1, knockout of miR-206 shows no obvious defect in muscle development [31]. These results demonstrate that the effect of single myogenesis miRNA on skeletal muscle development appear small. Usually, a mRNA possesses multiple miRNA binding sites for different miRNAs [32,33]. The possibility of functional compensation among myogenesis miRNAs, to some extent, can explain why knockdown or knockout of miR-1 do not result in obvious defects.

The inhibitor libraries for HTS makes it possible to explore miRNAs involved in cell growth, apoptosis, cell cycle and key signaling pathways in cell processes [34,35]. In the present study, a mini library of 88 dTuDs has been constructed within two days. Seven miRNAs were identified as AP-1 related by performing HTS. Although substantial downstream validations are required to validate their involvements in AP-1 signaling, miR-18b, -101 and -187 have been previously demonstrated to have functional roles in AP-1 regulatory network. AP-1 is an inducible heterodimeric protein composed of Jun, Fos, activating transcription factor and Jun dimerization protein subgroups of transcription factors [25]. Elevated miR-18b expression in human nasopharyngeal carcinoma accelerates cell growth, and knockdown of C-Jun causes a dramatic decrease of miR-18b expression [36]; AP-1 binds to miR-101 promoter and activates its expression where miR-101 can further suppress the c-Fos and subsequently attenuate the AP-1 signaling [37]; miR-187 is expressed at high level in ovarian cancer, and it inhibits the expression of disabled-2 tumor suppressor who can suppress tumorigenicity by reducing c-Fos expression [38,39]. Collectively, these findings revealed the important roles of miR-18b, -101 and -187 in AP-1 signaling. On the other hand, these validated miRNAs confirmed that our PCR-based method can accurately generate dTuDs. Moreover, as already discussed, dTuD vectors can be constructed at a greatly reduced cost. This method thus has great advantages in generating TuD miRNA inhibitor libraries.

## Conclusions

In summary, we designed a novel lentiviral vector dTuD with high specificity and efficiency in miRNA suppression *in vitro* and *in vivo*. We developed a method of two-step PCR amplification in combine with Golden Gate cloning strategy for dTuD vector construction. One important advantage of this method is that only two sets of short primers are required for a new dTuD, which can largely reduce the time and cost of TuD generation as well as increase inhibition efficacy.

## Supporting Information

S1 Fig The initial expression levels of miR-20a, -92a, -195 and -497 in 293A cells. (PDF)

S2 Fig The initial expression levels of miR-15a, -15b, -16, -107, -195, -424, -497, and -503 in 293A cells. (PDF)

S3 Fig The mRNA expression level of *CDC25A* or *CCNE1* in C2C12 cells transduced with dTuD-miR-322 or dTuD-miR-497. (PDF)

S4 Fig Body weight changes of mice injected with dTuD-miR-1 and dTuD-Ctrl from 2 to 8 weeks of age. (PDF)

S1 File Excel-based program dTuD_Primer_Design. (XLS)

S1 Table Primers used to amplify MBS-donor of dTuDs. (PDF)

S2 Table Sequences of TuD 3′stem-ccdB-TuD 3′stem within MBS-recipient vector and MBS-donor. (PDF)
