# Supplementary figures and images for: A PCR-Based Method to Construct Lentiviral Vector Expressing Double Tough Decoy for miRNA Inhibition
Source: PLoS One. 2015 Dec 1;10(12):e0143864. doi: 10.1371/journal.pone.0143864 (PMC4666662; doi:10.1371/journal.pone.0143864)

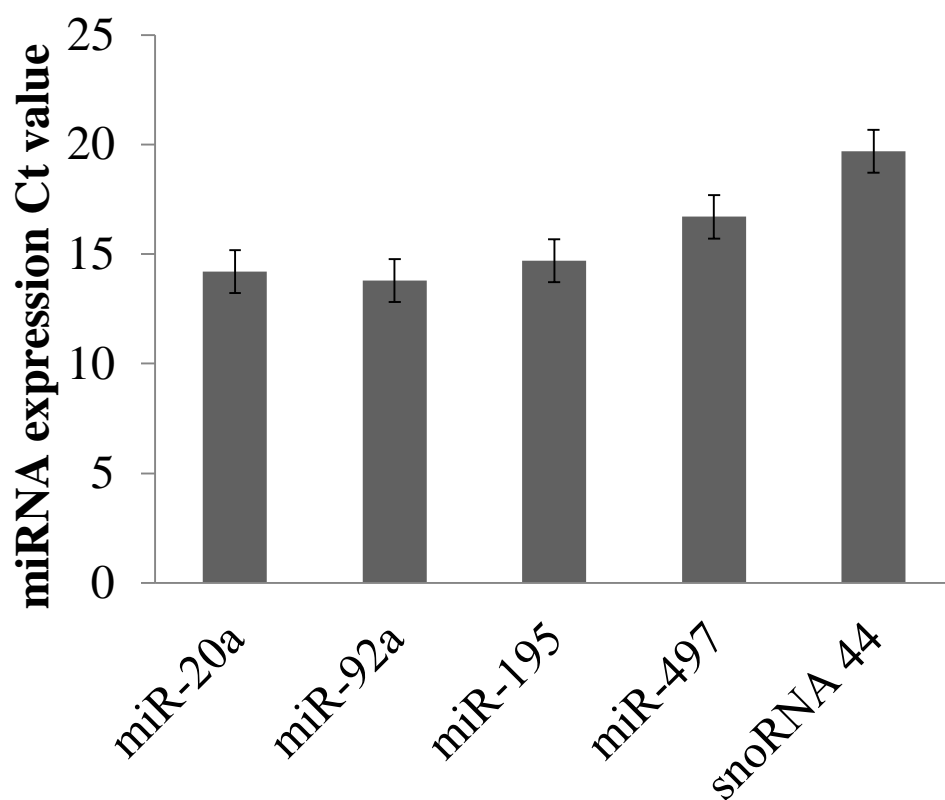

S1 Fig. The initial expression levels of miR-20a, -92a, -195 and -497 in 293A cells.

Supplement: S1 Fig — (PDF) [file pone.0143864.s001.pdf]

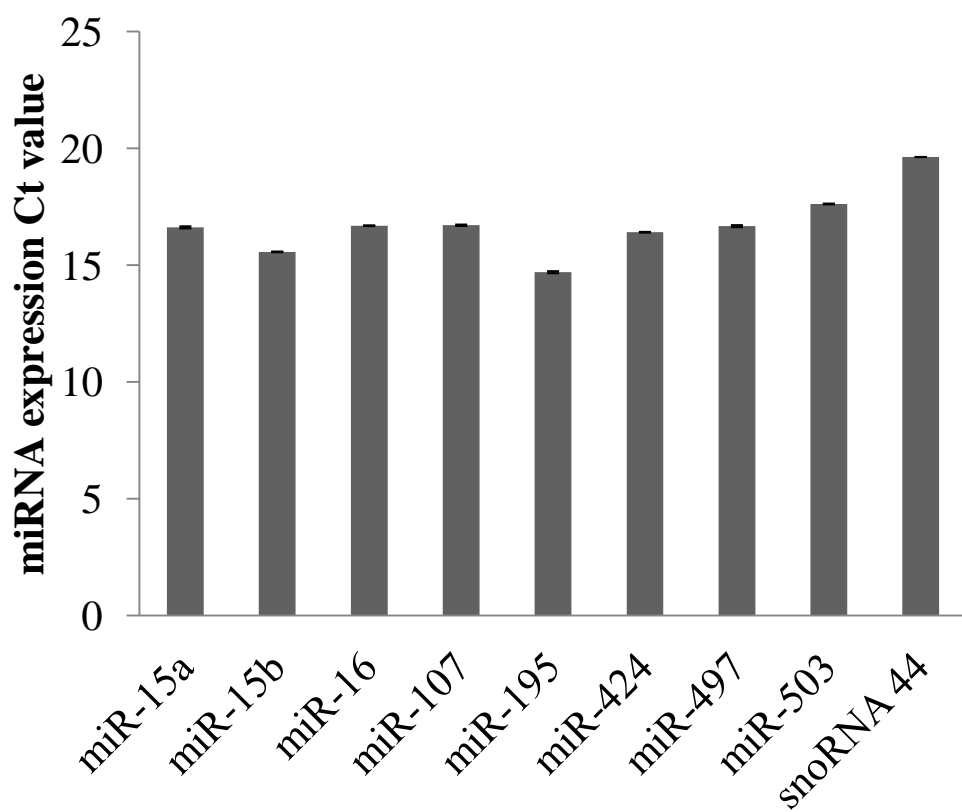

S2 Fig. The initial expression levels of miR-15a, -15b, -16, -107, -195, -424, -497, and -503 in 293A cells. Data are presented as the mean  $\pm$  SD (n=3).

Supplement: S2 Fig — (PDF) [file pone.0143864.s002.pdf]

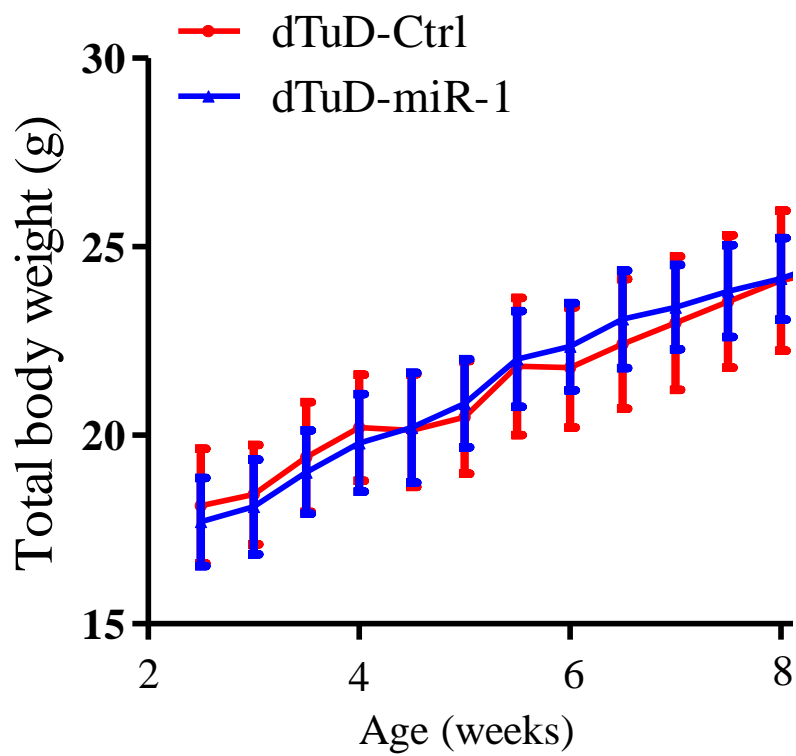

S4 Fig. Body weight changes of mice injected with dTuD-miR-1 and dTuD-Ctrl from 2 to 8 weeks of age.

Supplement: S4 Fig — (PDF) [file pone.0143864.s004.pdf]
