# Supplementary material for: A PCR-Based Method to Construct Lentiviral Vector Expressing Double Tough Decoy for miRNA Inhibition
Source: PLoS One. 2015 Dec 1;10(12):e0143864. doi: 10.1371/journal.pone.0143864 (PMC4666662; doi:10.1371/journal.pone.0143864)
Supplement: S3 Fig — (PDF) [file pone.0143864.s003.pdf]

A

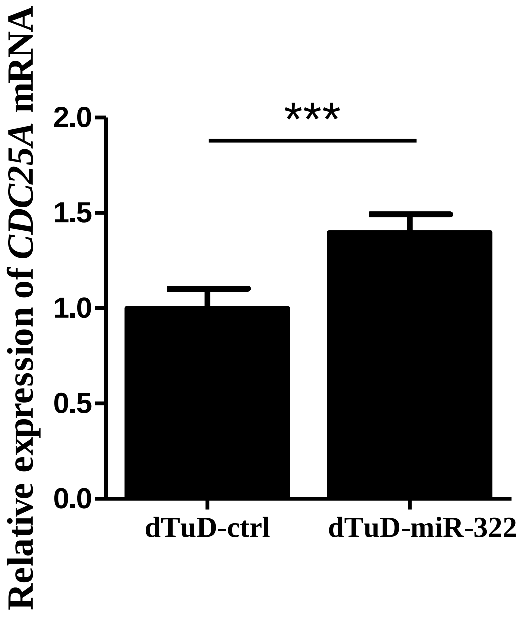

B

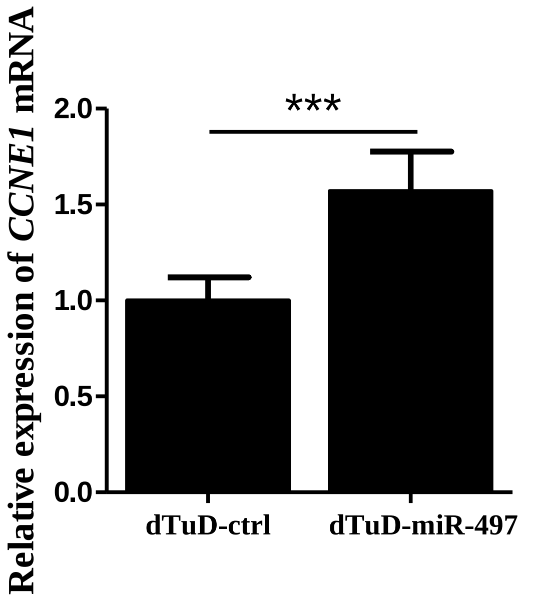

S3 Fig. The mRNA expression level of *CDC25A* (A) or *CCNE1* (B) in C2C12 cells transduced with dTuD-miR-322 or dTuD-miR-497.
