# Supplementary material for: A PCR-Based Method to Construct Lentiviral Vector Expressing Double Tough Decoy for miRNA Inhibition
Source: PLoS One. 2015 Dec 1;10(12):e0143864. doi: 10.1371/journal.pone.0143864 (PMC4666662; doi:10.1371/journal.pone.0143864)
Supplement: S1 Table — (PDF) [file pone.0143864.s006.pdf]

S1 Table. Primers used to amplify MBS-donor of dTuDs.

dTuD-ctrl-P1	5′-GTATTCTGTGACCAGAATACTTCTCACCGGGTGATCTTAAATCAGCTTGCTTGACTCTC-3′
dTuD-ctrl-P2	5′-GAATATAGGAATCTATATTCGGCCAAGCTGATTTAAGATCACCCGGTGACGGAACTTGA-3′
dTuD-ctrl-P3	5′-ACCGGTCTCTCTTCTCACCGGGTGATCTTAAATCAGCTTGCTTGTATTCTGTGACCAGA-3′
dTuD-ctrl-P4	5′-GTGGGTCTCATGGCCAAGCTGATTTAAGATCACCCGGTGACGGGAATATAGGAATCTAT-3′
dTuD-miR-223-P1	5′-GTATTCTGTGACCAGAATACTTCTGTCAGTTTGATCTTCAAATACCCCCTTGACTCTC-3′
dTuD-miR-223-P2	5′-GAATATAGGAATCTATATTCGGCGGGGTATTTGAAGATCAAACTGACACGGAACTTGA-3′
dTuD-miR-223-P3	5′-ACCGGTCTCTCTTCTGTCAGTTTGATCTTCAAATACCCCCTTGTATTCTGTGACCAGA-3′
dTuD-miR-223-P4	5′-GTGGGTCTCATGGCGGGGTATTTGAAGATCAAACTGACACGGGAATATAGGAATCTAT-3′
dTuD-miR-20a-P1	5′-GTATTCTGTGACCAGAATACTTCTAAAGTGCTTATCTATAGTGCAGGTAGCTTGACTCTC-3′
dTuD-miR-20a-P2	5′-GAATATAGGAATCTATATTCGGCCTACCTGCACTATAGATAAGCACTTTACGGAACTTGA-3′
dTuD-miR-20a-P3	5′-ACCGGTCTCTCTTCTAAAGTGCTTATCTATAGTGCAGGTAGCTTGTATTCTGTGACCAGA-3′
dTuD-miR-20a-P4	5′-GTGGGTCTCATGGCCTACCTGCACTATAGATAAGCACTTTACGGGAATATAGGAATCTAT-3′
dTuD-miR-92a-P1	5′-GTATTCTGTGACCAGAATACTTCTATTGCACTTATCTGTCCCGGCCTGCTTGACTCTC-3′
dTuD-miR-92a-P2	5′-GAATATAGGAATCTATATTCGGCCAGGCCGGGACAGATAAGTGCAATACGGAACTTGA-3′
dTuD-miR-92a-P3	5′-ACCGGTCTCTCTTCTATTGCACTTATCTGTCCCGGCCTGCTTGTATTCTGTGACCAGA-3′
dTuD-miR-92a-P4	5′-GTGGGTCTCATGGCCAGGCCGGGACAGATAAGTGCAATACGGGAATATAGGAATCTAT-3′
dTuD-miR-195-P1	5′-GTATTCTGTGACCAGAATACTTCTAGCAGCACAATCTGAAATATTGGCCTTGACTCTC-3′
dTuD-miR-195-P2	5′-GAATATAGGAATCTATATTCGGCGCCAATATTTCAGATTGTGCTGCTACGGAACTTGA-3′
dTuD-miR-195-P3	5′-ACCGGTCTCTCTTCTAGCAGCACAATCTGAAATATTGGCCTTGTATTCTGTGACCAGA-3′
dTuD-miR-195-P4	5′-GTGGGTCTCATGGCGCCAATATTTCAGATTGTGCTGCTACGGGAATATAGGAATCTAT-3′
dTuD-miR-497-P1	5′-GTATTCTGTGACCAGAATACTTCCAGCAGCACAATCTCTGTGGTTTGTACTTGACTCTC-3′
dTuD-miR-497-P2	5′-GAATATAGGAATCTATATTCGGCTACAAACCACAGAGATTGTGCTGCTGCGGAACTTGA-3′
dTuD-miR-497-P3	5′-ACCGGTCTCTCTTCCAGCAGCACAATCTCTGTGGTTTGTACTTGTATTCTGTGACCAGA-3′
dTuD-miR-497-P4	5′-GTGGGTCTCATGGCTACAAACCACAGAGATTGTGCTGCTGCGGGAATATAGGAATCTAT-3′
dTuD-miR-322-P1	5′-GTATTCTGTGACCAGAATACTTCCAGCAGCAATATCTTCATGTTTTGGACTTGACTCTC-3′
dTuD-miR-322-P2	5′-GAATATAGGAATCTATATTCGGCTCCAAAACATGAAGATATTGCTGCTGCGGAACTTGA-3′
dTuD-miR-322-P3	5′-ACCGGTCTCTCTTCCAGCAGCAATATCTTCATGTTTTGGACTTGTATTCTGTGACCAGA-3′
dTuD-miR-322-P4	5′-GTGGGTCTCATGGCTCCAAAACATGAAGATATTGCTGCTGCGGGAATATAGGAATCTAT-3′
dTuD-miR-1-P1	5′-GTATTCTGTGACCAGAATACTTCTGGAATGTAAATCTAGAAGTATGTATCTTGACTCTC-3′
dTuD-miR-1-P2	5′-GAATATAGGAATCTATATTCGGCATACATACTTCTAGATTTACATTCCACGGAACTTGA-3′
dTuD-miR-1-P3	5′-ACCGGTCTCTCTTCTGGAATGTAAATCTAGAAGTATGTATCTTGTATTCTGTGACCAGA-3′
dTuD-miR-1-P4	5′-GTGGGTCTCATGGCATACATACTTCTAGATTTACATTCCACGGGAATATAGGAATCTAT-3′

MBS of each miRNA is underline
