# Supplementary material for: A PCR-Based Method to Construct Lentiviral Vector Expressing Double Tough Decoy for miRNA Inhibition
Source: PLoS One. 2015 Dec 1;10(12):e0143864. doi: 10.1371/journal.pone.0143864 (PMC4666662; doi:10.1371/journal.pone.0143864)
Supplement: S2 Table — (PDF) [file pone.0143864.s007.pdf]

S2 Table. Sequences of 3′stem-ccdB- 3′stem within MBS-recipient vector.

> 3′stem-ccdB-3′stem within MBS-recipient vector : 3′stem-ccdB- 3′stem
5′-ATCGATAAAAAAAGACGGCGCTAGGAGAGTCTTCTGAGACGCATTAGGCACCCCAGGCTTTACACT<br>TTATGCTTCCGGCTCGTATAATGTGTGGATTTTGAGTTAGGATCGGGCGAGATTTTCAGGAGCTAAGGA<br>AGCTAAAATGGAGAAAAAAATCACTGGATATACCACCGTTGATATATCCCAATGGCATCGTAAAGAACA<br>TTTTGAGGCATTTCAGTCAGTTGCTCAATGTACCTATAACCAGACCGTTCAGCTGGATATTACGGCCTTT<br>TTAAAGACCGTAAAGAAAAATAAGCACAAGTTTTATCCGGCCTTTATTCACATTCTTGCCCGCCTGATG<br>AATGCTCATCCGGAGTTCCGTATGGCAATGAAAGACGGTGAGCTGGTGATATGGGATAGTGTTCACCCT<br>TGTTACACCGTTTTCCATGAGCAAACTGAAACGTTTTCATCGCTCTGGAGTGAATACCACGACGATTTC<br>CGGCAGTTTCTACACATATATTCGCAAGATGTGGCGTGTTACGGTGAAAACCTGGCCTATTTCCCTAAA<br>GGGTTTATTGAGAATATGTTTTTCGTgTCAGCCAATCCCTGGGTGAGTTTCACCAGTTTTGATTTAAACG<br>TGGCCAATATGGACAACTTCTTCGCCCCCGTTTTCACCATGGGCAAATATTATACGCAAGGCGACAAGG<br>TGCTGATGCCGCTGGCGATTCAGGTTCATCATGCCGTCTGTGATGGCTTCCATGTCGGCAGAATGCTTA<br>ATGAATTACAACAGTACTGCGATGAGTGGCAGGGCGGGGCGTAAAGATCTGGATCGGGCTTACTAAAA<br>GCCAGATAACAGTATGCGTATTTGCGCGCTGATTTTTGCGGTATAAGAATATATACTGATATGTATACCCG<br>AAGTATGTCAAAAAGAGGTGTGCTATGAAGCAGCGTATTACAGTGACAGTTGACAGCGACAGCTATCA<br>GTTGCTCAAGGCATATATGATGTCAATATCTCCGGTCTGGTAAGCACAACCATGCAGAATGAAGCCCGT<br>CGTCTGCGTGCCGAACGCTGGAAAGCGGAAAATCAGGAAGGGATGGCTGAGGTCGCCCGGTTTATTG<br>AAATGAACGGCTCTTTTGCTGACGAGAACAGGGACTGGTGAAATGCAGTTTAAGGTTTACACCTATAA<br>AAGAGAGAGCCGTTATCGTCTGTTTGTGGATGTACAGAGTGATATTATTGACACGCCCGGGCGACGGA<br>TGGTGATCCCCCTGGCCAGTGCACGTCTGCTGTCAGATAAAGTCTCCCGTGAACTTTACCCGGTGGTG<br>CATATCGGGGATGAAAGCTGGCGCATGATGACCACCGATATGGCCAGTGTGCCGGTATCCGTTATCGGG<br>GAAGAAGTGGCTGATCTCAGCCACCGCGAAAATGACATCAAAAACGCCATTAACCTGATGTTCTGGGG<br>AATATAAATGTCAGGCTCCGTTATACACAGCCATCGTCTCTGCCAACTTGAGCTGAGTCGCTTTTTTTG<br>AATT-3′

The restriction sites are underline: *Cla*I(ATCGAT), *Eco*RI (GAATTC) and *Bsm*BI (CGTCTC).
